# Characterization of *lamin* Mutation Phenotypes in *Drosophila* and Comparison to Human Laminopathies

**DOI:** 10.1371/journal.pone.0000532

**Published:** 2007-06-13

**Authors:** Andrés Muñoz-Alarcón, Maja Pavlovic, Jasmine Wismar, Bertram Schmitt, Maria Eriksson, Per Kylsten, Mitchell S. Dushay

**Affiliations:** 1 Department of Life Sciences, Södertörns högskola, Huddinge, Sweden; 2 Department of Biosciences and Nutrition, Karolinska Institute, Stockholm, Sweden; 3 Max-Planck-Institut für Hirnforschung, Abteilung Neurochemie, Frankfurt, Germany; 4 Department of Comparative Physiology, EBC, Uppsala University, Uppsala, Sweden; Institut Pasteur Korea, Republic of Korea

## Abstract

Lamins are intermediate filament proteins that make up the nuclear lamina, a matrix underlying the nuclear membrane in all metazoan cells that is important for nuclear form and function. Vertebrate A-type lamins are expressed in differentiating cells, while B-type lamins are expressed ubiquitously. *Drosophila* has two lamin genes that are expressed in A- and B-type patterns, and it is assumed that similarly expressed lamins perform similar functions. However, *Drosophila* and vertebrate lamins are not orthologous, and their expression patterns evolved independently. It is therefore of interest to examine the effects of mutations in lamin genes. Mutations in the mammalian lamin A/C gene cause a range of diseases, collectively called laminopathies, that include muscular dystrophies and premature aging disorders. We compared the sequences of lamin genes from different species, and we have characterized larval and adult phenotypes in *Drosophila* bearing mutations in the *lam* gene that is expressed in the B-type pattern. Larvae move less and show subtle muscle defects, and surviving *lam* adults are flightless and walk like aged wild-type flies, suggesting that *lam* phenotypes might result from neuromuscular defects, premature aging, or both. The resemblance of *Drosophila lam* phenotypes to human laminopathies suggests that some lamin functions may be performed by differently expressed genes in flies and mammals. Such still-unknown functions thus would not be dependent on lamin gene expression pattern, suggesting the presence of other lamin functions that are expression dependent. Our results illustrate a complex interplay between lamin gene expression and function through evolution.

## Introduction

The nuclear lamina is a matrix of intermediate filament proteins underlying the nuclear membrane in metazoan cells that contributes to nuclear form and strength, and affects chromosome behavior and cell differentiation [1], [2]. Vertebrate lamins are of two types; A-type lamins are expressed in differentiating cells, while B-type lamins are expressed ubiquitously. *Drosophila* has two lamin genes; *lamC* and *lamDm_0_* (hereafter called *lam*) that are expressed in A- and B-type patterns respectively [1]. The similarity of expression patterns and the presence of C-terminal CaaX motifs in similarly expressed lamin genes in *Drosophila* and vertebrates often has been taken as evidence of similar functions [3]–[8]. However, this does not reflect a shared evolutionary history. Molecular analyses have shown that *Drosophila* and vertebrate lamins all evolved from a single gene in a common ancestor [9]–[12]. The similar lamin gene expression patterns in these two lineages thus arose through convergent evolution, undoubtedly driven by functions which are dependent upon these expression patterns that are shared by protostomes and deuterostomes. Importantly, the independent evolution of lamin genes also means that some protein functions could map to either type of gene in different lineages – if they do not depend on the pattern of gene expression.

Mutations in human lamin genes lead to a range of human diseases collectively called laminopathies. Mutations in the human lamin A gene (*LMNA*) cause muscular dystrophies, type 2 Charcot-Marie Tooth disease, and premature aging diseases, among others [13]–[17]. No viable mutations in human lamin B genes were known until recently, when three single nucleotide mutations within the *LMNB2* locus were found in lipodystrophy patients [18], and a duplication of the chromosomal region containing *LMNB1* was correlated with human leukodystropy [8]. It remains a mystery how changes in proteins expressed in all cells selectively affect certain tissues and what molecular functions are performed by the differently expressed lamins.

A decade ago, a mutation in the ubiquitously expressed *Drosophila lam* was shown to cause flightlessness and impaired movement in surviving adults [19]. We set out to explore similarities between *lam* mutant phenotypes in *Drosophila* and laminopathic diseases in man. We have characterized the phenotypes of a series of *lam* mutations. For the first time, we report larval locomotion and muscle defects, possible premature aging, dominant phenotypes, and the susceptibility of *lam* phenotypes to enhancement and suppression by genetic background effects. Reductions in movement, premature aging, dominant phenotypes, and sensitivity to genetic background all are similar to diseases caused by *LMNA* mutations in man. Our findings highlight possible structure function discordance between lamin genes in *Drosophila* and mammals, and set the stage for a new, more nuanced view of lamin function in the light of the genes' evolutionary history.

## Results

**Figure 1:**
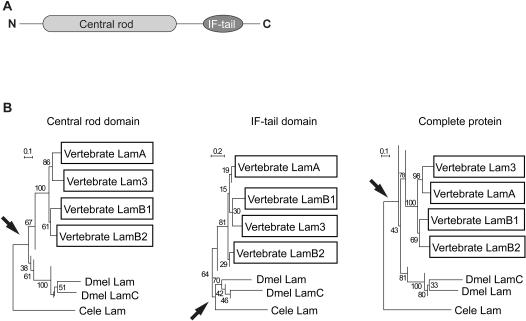
Comparison of lamin genes from different organisms. (A) Schematic diagram of a general lamin protein showing the central rod and IF-tail domains used in sequence comparisons. (B) Condensed cladograms showing the evolutionary relationship of 28 lamin genes, compared for the central rod domain, the IF-tail domain and full protein sequences. Protostome and deuterostome sequences group together rather than with different deuterostomic lamin groups. Note also how the central rod domain of the single C elegans lamin gene is equally related to deuterostome and protosome sequences, whereas its IF-tail domain groups with other protostomal sequences. The full C. elegans lamin sequence also occupies an intermediary position. Arrows indicate the root node of respective C.elegans sequences. Bootstrap values (1000 trials) are given as percent figures near nodes. See Materials and Methods for lamin designations, and Supplemental Figure S1 for full cladograms. Sequence alignments upon which these were based are available on request.

To study the evolutionary relationships among lamin genes we analyzed sequences from protostome and deuterostome species based on two conserved domains; the central rod, and the IF-tail, as well as whole proteins (Fig. 1A). The resulting alignments (Fig. S1, Supplemental Material) were used to construct three cladograms, all of which reveal that *Drosophila lam* and *lamC* are more closely related to each other than to either type of vertebrate lamin (Fig. 1B). Both *Drosophila* lamin domains and whole proteins all clearly grouped together and away from deuterostomes.

**Table 1:** *lam* lethality

	*lam* ^P^	*lam* ^D395^	*lam* ^D395^/Df	*lam* ^04643^	*lam* ^G262^	wild type
larval lethality	>99.5*	41	39	46	17	7
pupal lethality	<0.5*	58	55	54	10	10
adult escapers	<0.5*	1	6	10	73	83

Stages of lethality for different *lam* alleles. 200 larvae of each genotype were selected and scored for survival as described in Materials and Methods. Numbers refer to the percentage of animals that died during larval and pupation stages, and the percentage of adult escapers that emerged. Extensive counts of larval emergence did not reveal marked embryonic lethality for any of the *lam* alleles (data not shown). Wild type flies were *w* ^1118^ (Oregon R). Our observing only 83% wild type adult survival compared to expectations of 99% could be explained by culture conditions or trauma during handling, but as all genotypes were handled similarly, this did not bias results. Note the slight difference between *lam* ^D395^ homozygotes and *lam* ^D395^/Df (2L) cl-h1 transheterozygotes, which is discussed in the text. * No *lam* ^p^ pupae were observed in this experiment, but 5 *lam* ^p^ larvae survived to pupate from a much larger pool in our pupariation height experiment, shown in Figure 3B.

The two domains of the single *C. elegans* lamin behaved differently. The filament domain occupied an intermediate position between protostomes and deuterostomes, similar to the whole protein, whereas the IF-tail domain grouped with other protostome IF-tail domains. The significance of this result is not clear, but it has been noted that the *C. elegans* lamin evolved rapidly [10], [20]. It also may be relevant that *C. elegans* is the only metazoan with a sequenced genome that has only a single lamin gene (see Discussion).

**Figure 2:**
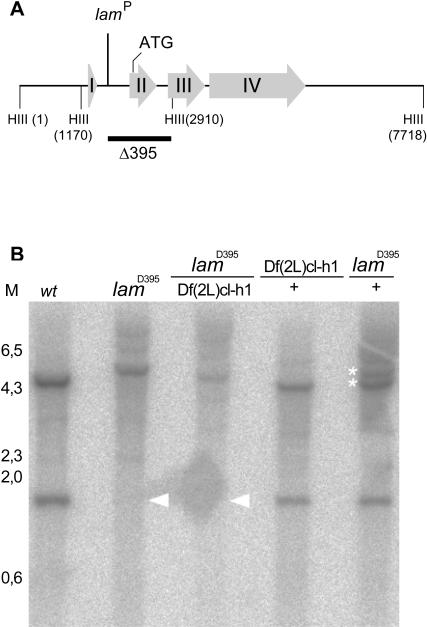
Molecular characterization of null allele *lam* ^D395^. (A) Schematic drawing of the lam locus. Exons are presented as large grey arrows, and relevant restriction sites and the translation start site are indicated. The site of insertion of the P element responsible for the *lam* ^P^ mutation is shown, and the bar underneath indicates the extent of the *lam* ^D395^excision. (B) Southern blot of genomic DNA from the genotypes indicated above each lane. The white arrowheads mark the band corresponding to the second exon lacking in *lam* ^D395^ homozygotes and *lam* ^D395^/*Df (2L) cl-h1* transheterozygotes. The white asterisks mark the 4.8 kb HinDIII band resulting from the wild type chromosome (lower) and the band from the mutant chromosome (upper), which is larger due to the deletion of the HinDIII (2910) site.

The lack of orthology between *Drosophila* and mammalian lamin genes means that while some important functions are almost certainly performed by lamin genes with comparable expression patterns in different species, this does not necessarily extend to all lamin gene functions. We therefore initiated studies on *Drosophila* lamin gene mutants. A *lamC* amorphic allele delayed development through embryonic/larval stages and caused complete pre-metamorphosis lethality, but showed no other developmental effects [6]. In contrast, the *lam* ^P^ mutation caused flightlessness and reduced movement in surviving (escaper) adults [19]. We studied four different *lam* alleles; *lam* ^P^, *lam* ^D395^, *lam* ^04643^, and *lam* ^G262^. The *lam* ^D395^ allele was generated by excision of the P-element insertion responsible for the *lam* ^P^ mutation [19], and we confirmed it is a null allele by showing the *lam* ^D395^ deletion removes exon II containing the translation start codon (Fig. 2). Thus *lam* ^D395^ is in the same category as the *lam* null alleles reported by Osouda [7].

### *lam* mutations likely cause dominant fitness effects

The lethality of *lam* mutations occurred during larval and pupal stages (Table 1), similar to that reported by Osouda *et al* [7]. We found greater and earlier lethality in *lam* ^D395^ homozygotes than in *lam* ^D395^/*Df(2L)cl-h1* transheterozygotes (Table 1). This suggested the presence of other genetic aberrations in the *lam* ^D395^ stock, since both homozygous and transheterozgyous animals were equally null for *lam* (Fig. 2B). To avoid interference with our characterization of *lam* phenotypes, we therefore employed *lam* ^D395^/*Df(2L)cl-h1* animals as *lam* nulls whenever possible.

Cultures of the four *lam* alleles produced unequal amounts of escapers (Table 1). Surprisingly, we saw no adults from the *lam* ^P^ stock where we expected 5–10% escapers [7], [19]. To confirm that the lethality in this *lam* ^P^ stock mapped to the *lam* gene, we remobilized the responsible P element and recovered viable and fertile excisions (data not shown). This showed that the P element insertion was the specific cause of lethality in the *lam* ^P^ stock, but did not explain the absence of escapers.

We then suspected that this might be due to an accumulation of genetic modifiers. This happens in *Drosophila* mutants with dominant effects on fitness (*e.g*. [21]), and this would also explain the greater lethality of *lam* ^D395^ homozygotes compared to *lam* ^D395^/*Df(2L)cl-h1* heterozygotes. We therefore randomized the genetic background of the *lam* ^P^ stock by iterated outcrosses to wild type. After six generations, we backcrossed *lam* ^P^/+ siblings together, and then we recovered adults with rough eyes and feeble movement, as *lam* ^P^ escapers had been described [19]. The reappearance of escapers in the outcrossed stock was consistent with the accumulation of genetic modifiers in our original stock and suggested that *lam* ^P^ has dominant effects on fitness (see Discussion). We continued outcrossing for another four generations (10 generations in all), and used an outcrossed *lam* ^P^_(oc)_ stock for subsequent experiments.

### Locomotor phenotypes in adult *lam* mutants are recessive

**Figure 3:**
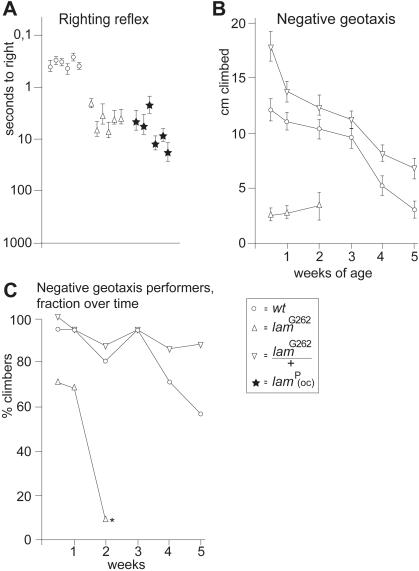
Adult behavior of *lam* mutants. (A) Righting reflex plotted on a log scale. Each symbol represents the mean of six trials for an individual adult, with error bars showing SEM. (B) Negative geotaxis was plotted as a function of genotype and age. Each point represents 20 – 35 adults, with error bars showing SEM. Data from males and females were pooled. (C) The percentage of living adults that climbed in the negative geotaxis assay, plotted as a factor of age. Homozygous *lam* ^G262^ mutants all died before three weeks of age.

We assessed *lam* adult behavior by monitoring the ability of *lam* ^04643^ and *lam* ^G262^ escapers to regain an upright position after being flipped on their backs (righting reflex) [22]. Of several hundred *lam* ^04643^ escapers that emerged, only one fly could be tested, while all of the others died too soon, or got stuck to the food. Thus, *lam* ^04643^ fell on the border of measurability, and the single testable fly took significantly longer to right itself than any of the other *lam* escapers. There were more testable *lam* ^G262^ escapers. These adults were slower to right themselves than controls (p<0.05 Fig. 3A). Outcrossed *lam* ^P^ escapers performed similarly to *lam* ^G262^ (Fig. 3A). Note that the performance of these alleles is not directly comparable because neither *lam* ^G262^ or *lam* ^04643^ escapers were from outcrossed stocks, so their righting reflex phenotypes were almost certainly enhanced by genetic modifiers. Regardless, all *lam* allele escapers righted slower than controls (Fig. 3A), and this phenotype was recessive, as heterozygous adults performed indistinguishably from controls (data not shown).

We also monitored adult locomotor activity by scoring *lam* ^G262^ negative geotaxis. These flies performed significantly less well (p<0.05) than *lam* ^G262^/+ siblings, which were indistinguishable from wild-type (Fig. 3B). As performance declines with the age of the fly, we assayed the behavior of adults up to five weeks of age. Performance of *lam* ^G262^/+ and control flies declined similarly, while homozygotes did not show any decrease beyond their initial low levels over the two weeks they survived, probably because they climbed too little to show any further decrement (Fig. 3B). We also assessed negative geotaxis by plotting the relative number of adults that climbed. The fraction of wildtype and *lam* ^G262^/+ flies that performed decreased over time. This was also true for *lam* ^G262^ flies, but the fraction of mutants that climbed was lower than age matched controls, and the decrease was much more dramatic for mutant flies, approaching zero at two weeks of age. (Fig. 3C).

### Dominant *lam* effects on pupariation height, and recessive effects on larval locomotion

**Figure 4:**
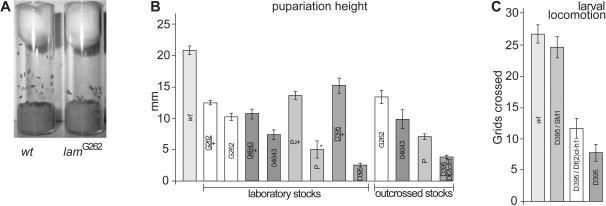
Larval behavior of *lam* mutants. (A) Vials showing the difference in pupariation height between wild type at left, and *lam* ^G262^at right. (B) Chart of pupariation height in mm. Error bars correspond to SEM. Differences between *lam* ^D395^ and *lam* ^D395^/*Df(2L)cl-h1*, and outcrossing are described in the text. Sample sizes; wt (*w* ^1118^) = 163, G262/+(*lam* ^G262^/+) = 114, G262 (*lam* ^G262^) = 133, 04643/+(*lam* ^04643^/+) = 40, 04643 (*lam* ^04643 ^) = 45, P/+(*lam* ^P^/+) = 105, P (*lam* ^P^) = 5, D395/+(*lam* ^D395^/+) = 74, D395 (*lam* ^D395^) = 33, G262 outcrossed = 39, 04643 outcrossed = 19, P outcrossed = 94, and D395/Df = 87. The asterisk marks *lam* ^P^ laboratory strain, where we had to screen a very large number of larvae to find 5 pupae to measure - see also the footnote to *lam* ^P^ laboratory strain lethality in Table 1. (C) Larval locomotion is shown for the indicated genotypes as the number of 5 mm squares crossed in 5 minutes (see Materials and Methods). Error bars correspond to SEM. In all cases, n = 30.

During this work, we noted that *lam* larvae pupariated lower on the sides of the vials than controls did (Fig. 4A), suggesting that *lam* larvae moved less. All four *lam* alleles pupariated significantly lower than wild type (p<0.05, Fig. 4B). Pupariation height has been correlated to larval locomotion [23], but it is also affected by humidity and crowding [24]. To confirm that lower pupariation height reflected less larval locomotion, we measured the movement of wandering stage *lam* ^D395^/*Df(2L)cl-h1* larvae. These null mutant larvae moved significantly less than controls or *lam*/+ heterozygotes (p<0.01, Fig. 4C). Thus lowered pupariation height did reflect less larval movement, and *lam* mutations reduce both larval and adult locomotion.

To test if genetic modifiers also affected pupariation height, we scored *lam* ^D395^/*Df(2L)cl-h1* transheterozygotes, partially out-crossed *lam* ^04643^ and *lam* ^G262^ (see Materials and Methods), and outcrossed *lam* ^P ^larvae. As shown in Fig. 4B, outcrossing significantly improved pupariation height for all four alleles (p<0.05), while the *lam* ^04643^, *lam* ^G262^, and null alleles maintained their rank order. Unlike the adult phenotypes and larval locomotion, *lam* effects on pupariation height were dominant, as *lam*/+ heterozygotes pupariated at heights intermediate between *lam* homozygotes and wild type. This discrepancy does not alter the value of pupariation height as a measure of larval locomotion, but probably reflects the influence of additional factors on pupariation height.

### Loss of LAM function causes minor lesions in muscle

**Figure 5:**
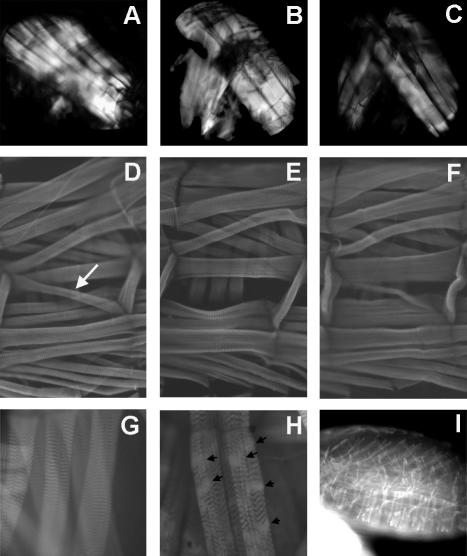
Muscle histology. (A–C) Adult indirect flight muscle morphology. Thoraces were cleared and examined by polarized light microscopy. (A) Wild type w1118. (B) lamG262 and (C) lam04643. Note that this technique does not allow fine focus, but permits gross assessment of muscle bulk and organization through entire thoraces. (D–F) Abdominal segmental muscle fibers labeled by rhodamine-conjugated phalloidin. (D) Wild type w1118. Muscle 5 is indicated by a white arrow. (E–F). lam04643 showing absence (E) or misinsertion (F) of muscle 5. (G) Higher magnification of phalloidin-stained w1118 larval body wall muscles showing regular patterns. (F) lam04643 - fine structure defects are highlighted by arrowheads. Similar defects are seen in other alleles (data not shown). (I) Phalloidin-stained late stage lam04643 embryo demonstrating normal muscle organization and form.

Flightlessness, inability to right, and reduced geotaxis in adults, and less locomotion in larvae all could be caused by muscular defects, so we examined muscle in *lam* mutants. In contrast to muscular dystrophies that cause gross changes in muscle structure [25], muscles from *lam* mutant animals showed only minor changes. Examination of adult indirect flight muscles in cleared whole adult thoraces by polarized light microscopy did not reveal gross differences between *lam* adult escapers and controls in muscle bulk, placement, or organization (Fig 5A–C). Neither were great changes in muscle size or organization detected in *lam* mutant embryos (Fig 5I). However, examination of larval abdominal body wall hemisegments revealed that muscle 5 (numbering scheme of [26] and indicated by a white arrow in Fig 5D) was often absent (Fig 5E) or slightly reduced in size and misinserted in *lam* mutants (Fig 5F). In addition, fine structure defects in fibrillar organization were also found in *lam* larvae that were never observed in age-matched control larvae (Fig 5G–H). Neither the loss or misinsertion of muscle 5 nor fine structure defects could account for the locomotor phenotype of *lam* larvae, but the prevalence of fine fibrillar defects correlated with the strength of behavioral phenotypes between different alleles. This suggests that some defects in *lam* larval muscles might develop with age, use, or progressive loss of protein translated from maternal *lam* mRNA [7], [27].

### *lam* over-expression kills in a levels and tissue specific manner

The lethality and adult phenotypes of *lam* mutations have been rescued by transformation with a *lam* genomic clone [7], [19]. We chose to over-express *lam* and *lamC* cDNA constructs using the two component GAL4-UAS system [28], for several reasons. We wanted to test whether we could rescue *lam* mutant phenotypes by expressing lamin proteins in specific tissues. We also wanted to test the phenotypic consequences of *lam* and *lamC* over-expression. Finally, we sought to test the molecular mechanisms underlying similarities between laminopathic phenotypes in flies and humans — despite the different patterns of gene expression — by transforming flies with cDNA constructs of human *LMNA* (pUAST-LA) and Progerin (pUAST-LAΔ150). Progerin is the mutant form of lamin A present in children with the premature aging disease Hutchinson-Gilford Progeria syndrome [29], [30]. *Drosophila* UAS::*lam* and both human UAS::*LMNA* constructs were lethal when expressed ubiquitously in wild type animals. We combined different UAS::*lam* insertions with ubiquitous GAL4 drivers of different strengths and reared animals at 25°C (less-) and 29°C (greater expression) (Table 2). Only the weaker UAS::*lam* insertion with the weakest ubiquitous driver (*arm*) at the lower temperature produced any adults, which were indistinguishable from wildtype flies. Lethality also resulted when UAS::*lam* expression was driven in the mesoderm alone. In contrast, expression in hemocytes, or in the nervous system driven by nrv2::Gal4 had no noticeable effect. We observed similar phenotypes with all GAL4 drivers in both wild type and *lam* mutant backgrounds (data not shown).

**Table 2:** Overexpression lethality of *lam* cDNA constructs

		*lam* 354		*lam* 357	
**GAL4 Driver**	**Tissue**	25°C	29°C	25°C	29°C
da	ubiquitous	lethal	lethal	lethal	lethal
arm	ubiquitous	viable	lethal	lethal	lethal
P{GawB}how^24B^	mesoderm	lethal			
P{GawB}C23	mesoderm	lethal			
hemese	hemocytes	viable			
nrv2	nerves	viable			

The lethality of overexpressing *lam* cDNA ubiquitously or in mesoderm, but not in hemocytes or neurons. The GAL4 drivers used were da - daughterless, strong ubiquitous expression; arm - armadillo, weaker ubiquitous expression, P{GawB}^how24B^- mesoderm; P{GawB}C23 - transverse muscles; hemese - hemocytes [49]; and nrv2 - nervana, nervous system. Expression was driven by the indicated GAL4 drivers, and cultures were held at 25°C and 29°C. Viability was scored as the survival of any adults from ≥70 embryos. Note that ubiquitous *lam* overexpression was lethal in every case except for the presumably weaker expressed cDNA construct driven by the weak *arm* ubiquitous GAL4 driver at the lower temperature (less GAL4 activity).

Mammalian cells are also sensitive to the levels of lamin gene expression. In addition to the laminopathies caused by loss-of-function mutations, disease and changes in cultured cells are associated with overexpression of both *LMNA* [31]–[33] and *LMNB1* [8]. Recently, it was reported that overexpression of human *LMNB1* and *Drosophila lam* in the developing eyes and central nervous system of flies controlled by the GMR and ELAV GAL4 drivers caused severe malformation and lethality [8]. We obtained similar results using the same drivers and the more potent of our two *lam* cDNA constructs (data not shown). This indicated that these effects, as opposed to the lack of discernable phenotypes with the *nrv2* GAL4 driver, are due to high levels of *lam* expression, as well as possible differences in expression pattern or timing.

While mammalian cells are sensitive to the levels of both *LMNA* and *LMNB1* gene expression, *Drosophila* are uniquely sensitive to overexpression of *lam*. Whereas expression of mutant forms of *lamC* have been shown to cause phenotypes in Drosophila [5], [6], overexpression of a wildtype *lamC* cDNA construct caused no noticeable phenotypes, and adults were fully fertile when using the same drivers and conditions that killed flies bearing *lam* constructs (data not shown, but see Table 2 for the GAL4 drivers). Thus proteins coded by the two *Drosophila* lamin genes function differently.

## Discussion

Analyses of lamins from different taxa have shown that protostome and deuterostome lamin genes split and evolved similar patterns of expression independently [9]–[12]. However, lamins are modular proteins with two distinct domains; the filament and IF-tail, and domain-limited sequence similarities could have gone undetected. To test this, we analyzed lamin domain sequences and obtained results very much like whole protein comparisons; *Drosophila* and mammalian lamin genes evolved independently, and similar expression patterns evolved convergently. This points to still-unknown expression-dependent lamin gene functions being performed by similarly expressed genes in the different lineages. Yet not necessarily all lamin gene functions are expression-dependent.

We have characterized the phenotypes of *Drosophila lam* mutants. We describe lethal stage, and escaper adult flightlessness, negative geotaxis, and righting reflex for an allelic series of *lam* loss-of-function mutants. We also describe for the first time *lam* effects on larval locomotion and muscle fine structure, and dominant effects on pupariation height and fitness. The movement phenotypes of larvae and surviving *lam* adults recall effects of mutations in the human *LMNA* gene. In contrast, mutations of the *lamC* gene are prepupal lethal [6]. The locomotor effects of mutations in the ubiquitously-expressed *Drosophila lam* gene thus appear similar to some of the effects of mutations in the human *LMNA* gene. Combined with lamin genes' evolutionary relationship, this suggests that some of the unknown molecular functions underlying these effects do not depend on a restricted lamin gene expression pattern and have segregated to differently expressed lamin genes in vertebrate and invertebrate lineages.

The idea that lamin functions partitioned differently in different species is also supported by the fact that not all metazoans express two types of lamins. Egg-laying vertebrates have a third lamin type; lamin LIII, preferentially expressed in the egg [10]. *C. elegans* has only one lamin gene, and this gene must perform all the necessary functions of different lamins in other species. This is supported by the findings of Haithcock et al., who showed that loss of *C. elegans* lamin reduced lifespan and caused nuclear changes associated with aging [34]. Thus, in *C elegans* loss of the ubiquitously expressed lamin results in laminopathy-like premature aging. We note that the behavior of young *lam* escaper adults were like those of aged wildtype flies [35]. Thus it is also possible that *lam* mutations could cause a form of premature senescence in *Drosophila*.

We do not yet know in which tissues loss of lamin protein function is responsible for *lam* phenotypes. The minor effects we could detect in muscle point to a possible neurological involvement, and this is supported by changes in the central nervous system [19] and underdevelopment of ventral ganglia in *lam* mutants [7]. We attempted to address this question by expressing *lam* cDNA in specific tissues, but found that overexpression of both *Drosophila* and human cDNA constructs was lethal, with mesoderm, or subsets of mesodermal tissues particularly sensitive to Lamin protein levels (Table 2). Strong overexpression of *lam* cDNA in developing eye and nervous system was also lethal, as reported by [8] and (data not shown). The levels of Lamin protein were not measured by Western blot because we do not know which cells are responsible for the lethality, and previous attempts to correlate whole-animal Lamin levels with lethality were inconclusive [7]. Experiments are currently underway to address in which tissues loss of lamin protein cause lethality with somatic null clones.

The recovery of escaper adults from the *lam* ^P^ stock after outcrossing, the greater viability of *lam* ^D395^/*Df(2L)cl-h1* transheterozygotes compared to *lam* ^D395^ homozygotes, and the changes in pupariation height of two other *lam* alleles after outcrossing all point to accumulation of genetic modifiers in *lam* fly stocks. These are not responsible for the mutant phenotypes themselves: these map to *lam* as demonstrated by their expression in all tested *lam* alleles that were independently generated in different genetic backgrounds, persistence through outcrossing, and by the recovery of viability with the precision excision of the P element responsible for the *lam* ^P^ allele. The presence of other, non-*lam* genetic factors that modify *lam* phenotypes compels three conclusions; i) *lam* mutations have a dominant effect on the fitness of heterozygous animals (driving the accumulation of modifiers), ii) this phenotype is susceptible to suppression by other genes not linked to *lam*, and iii) fitness-effect suppressing genes enhance other *lam* phenotypes. This is similar to man, where genetic modifiers are indicated by finding closely related individuals with identical *LMNA* mutations showing differences in disease severity [36]–[39]. The genetic dominance of *lam* effects on *Drosophila* fitness and pupariation height is similar to the dominance of *LMNA* mutations in man that cause Autosomal Dominant Emery-Dreifuss muscular dystrophy [40].

In summary, we conclude that similarly expressed *Drosophila* and mammal lamins are not orthologous, and changes in gene sequence or expression of *Drosophila lam* cause locomotor defects and possibly premature aging. These effects are similar to effects of mutations in the mammalian *LMNA* gene. We do not suggest that *Drosophila lam* mutants are a model for mammalian laminopathies. Rather, our findings bring two questions into focus. i) What are the molecular functions underlying lamin mutation effects, and ii) What other molecular functions are dependent on lamin gene expression pattern and led to their independent evolution in both protostomes and deuterostomes? Further studies on flies and other organisms will be required to address these questions. As so much is inferred regarding structural, functional and expression pattern homologies by labeling lamins A-type or B-type, we suggest that these terms only be used for vertebrate lamins as they are not fully meaningful for describing lamins in protostomes.

## Materials and Methods

### Fly strains

The generation of the *lam* ^P^ allele has been described by Lenz-Böhme [19].

The *lam* ^04643^ allele results from a P element insertion into the first intron, independent of the *lam* ^P^ insertion. This stock was obtained from the Bloomington Drosophila Stock Center. A partially outcrossed *lam* ^04643^ stock for pupariation height experiments (Fig. 4B) was generated by several generations of crossing *lam* ^04643^/CyOACTGFP to CyOACTGFP/*In(2LR)Gla* and selecting *Gla*+ progeny.

The *lam* ^G262^ allele [41] is a protein-trap insertion resulting in the expression of a lamin-GFP fusion protein. Our results show this allele acted as a weak hypomorph. These flies were the generous gift of William Chia. A partially outcrossed *lam* ^G262^ stock was generated similarly to *lam* ^04643^, above.

The *lam* ^D395^ allele is a null generated by excision of the P element from *lam* ^P^ (J.W. and B.S.).

*Df(2L)cl-h1*, a deficiency uncovering *lam* was obtained from the Bloomington Stock Center, as was *w* ^1118^, which was used as a wild type control.

### Viability

Homozygous *lam* larvae were selected as non-fluorescent progeny of *lam*/*SM1*ActGFP heterozygous parents. *ca* 200 larvae per genotype were put into vials (50 larvae per vial). Vials were kept at 25°C, and the number of pupae were counted within a period of 1–2 weeks. Emerging escaper adults were counted for a period of up to two weeks after pupariation.

### Behavioral assays

Negative geotaxis was measured by the method of Goddeeris [42]. Briefly, one-day-old flies were anaesthetized with CO_2_, sorted singly into measuring cylinders, and allowed to recover for 10 minutes. Flies were tapped to the bottom, and the height climbed within 10 seconds was recorded.

Righting reflex was measured as described by Leal [22]. Individual one-day-old flies were gently flipped onto their backs, and the time it took to right themselves to a standing position was clocked by stopwatch. Each fly was tested 6 times in one day.

Pupariation height. Fifty 2nd to 3rd instar larvae were placed in vials to pupariate, and the height of pupae above the food surface was measured. Pupae in contact with the food were not scored.

Larval locomotion was scored by the method of Yang [43], [44].

### Muscle histology

Indirect flight muscles from three day-old flies were examined by removing heads and abdomens, dehydrating in an ethanol series, and leaving in methyl salicylate overnight. Thoraces were mounted in methyl salicylate and studied under polarized light.

Larval body wall preparations were performed by dissecting larvae in PBS (phosphate buffered saline, pH 7,2) 200 µM EGTA, washing in PBS, fixing in 4% paraformaldehyde, and washing with PBS 0.1% Triton. Samples with stained in 1∶100 phalloidin-rhodamine 30 minutes at 4°C, washed 3 times in PBS, and mounted in glycerol.

Larval muscles were prepared for fine fibrillar pattern examination as follows: ten 3rd instar larvae were put in 65°C water for 5–10 sec. Then larvae were dissected in calcium-free Ringer's buffer, leaving muscles attached to the body wall, fixed in 4% formaldehyde, washed in 80% ethanol, and set in PBS. Muscles were stained with FITC-conjugated Phalloidin in PBS for 40 minutes at room temperature. Preparations were examined in a Zeiss M2 FLS microscope equipped for fluorescence.

Embryonic muscles were examined by the method of Hidalgo [45]. Briefly, dechorionated, devitellinized embryos were stained in 1∶100 diluted phalloidin-rhodamine 40 minutes at RT, and rinsed in PBS 0.2% Tween.

### Statistics

Statistical analysis was performed in Microsoft Excel using T-tests assuming unequal variance. The confidence interval was set to 95% for each of the tests.

### Southern analysis

DNA from 25 – 50 larvae was isolated according to Hamilton [46], with the addition of a 100µg/ml Proteinase K step overnight after RNAse A treatment. 8 µg DNA from each genotype was digested overnight at 37°C with HindIII and separated by electrophoresis on a 0.6% agar gel. Blotting onto Hybond N+ membranes, hybridization, and detection was according to Sambrook [47]. The probe was made from the LD38055 cDNA clone (obtained from BDGP) using the random prime-kit (GE Healthcare Amersham) and Redivue 32P-dCTP (GE Healthcare). The probe was used at 10^5^–10^6^ dpm ml^−1^ specific activity.

### Amino acid sequence comparisons

ClustalX (1.81) for Macintosh software was used, at the standard setting, to align full-length sequences for nuclear lamins, for bootstrapping calculations and for building phylogenetic trees, which were plotted using the NJplot software. Trees were corrected for multiple substitutions. See table S1 for sequence accession numbers.

### Cloning UAS constructs

Full-length cDNA clones LD38055 (*lam*) and LD31805 (*lamC*) were obtained from the BDGP, and inserts were transferred into the pUAST-vector using directional cloning via the *Eco* R1 and *Xho* 1 restriction enzymes. The pUAST-LA and pUAST-LAΔ150 expression vectors were created by subcloning human cDNA of lamin A (*LMNA*) and progerin (LAΔ150) from the pET24 –LA and pET24-LAΔ150 vectors [48]. pET24-LA and pET24-LAΔ150 were digested with BamHI, blunt ended, and digested with NotI. Following gel purification the released cDNA fragments were ligated to the pUAST expression vector that had been digested with EcoRI, blunted, and digested with NotI. Clones were screened by PCR (5′-gcaacaagtccaatgaggacca-3′ and 5′-gtcccagattacatgatgc-3′) and verified by restriction digests (NotI and AscI) and sequencing (5′-cgctccttggctactgagtc-3′, 5′-gtggaaggcacagaacacct-3′, and 5′-gcaacaagtccaatgaggacca-3′) (data not shown).

Transgenic animals were generated by the Umeå transgenic facility. For all experiments, several independent single inserts of each construct on the third chromosome were used.

## Supporting Information

Table S1 Accession numbers for the sequences used in Fig 1B and Fig. S1
(0.04 MB DOC)

Figure S1 Lamin phylogenetic comparisons. The three cladograms described in the text and presented in a condensed format in Fig. 5B are presented here in full.
(0.17 MB PDF)
