# Supplementary material for: Characterization of lamin Mutation Phenotypes in Drosophila and Comparison to Human Laminopathies
Source: PLoS One. 2007 Jun 13;2(6):e532. doi: 10.1371/journal.pone.0000532 (PMC1885830; doi:10.1371/journal.pone.0000532)
Supplement: Table S1 — Accession numbers for the sequences used in Fig 1B and Fig. S1 (0.04 MB DOC) [file pone.0000532.s001.doc]

**Table S1.**

Accession numbers for the sequences used in Fig 1B and Fig. S1.

| Designation | Species | Sequence accession number |
| --- | --- | --- |
| Aaeg_Lam48669 | *Aedes aegypti* | EAT48699 |
| Aaeg_Lam45279 | *Aedes aegypti* | EAT45279 |
| Amel_Lam | *Apis mellifera* | XP_396670 (further edited by eye from the NW 623060 contig) |
| Blan_Lam | *Branchiostoma lanceolatum* | CAC13104 |
| Cele_Lam | *Caenorhabditis elegans* | CAA52188 |
| Dmel_LamC | *Drosophila melanogaster* | NP_523742 |
| Dmel_Lam | *Drosophila melanogaster* | Lam, P08928 |
| Drer_LamA | *Danio rerio* | NP_694503 |
| Drer_LamB1 | *Danio rerio* | NP_694504 |
| Drer_LamB2 | *Danio rerio* | CAB41015 |
| Drer_LamL3 | *Danio rerio* | NP_694505 |
| Ggal_LamA | *Gallus gallus* | NP_990618 |
| Ggal_LamB1 | *Gallus gallus* | NP_990617 |
| Ggal_LamB2 | *Gallus gallus* | NP_990616 |
| Ggal_LamL3 | *Gallus gallus* | XP_413842 |
| Hsap_LamA | *Homo sapiens* | P02545 |
| Hsap_LamB1 | *Homo sapiens* | AAH12295 |
| Hsap_LamB2 | *Homo sapiens* | NP_116126 |
| Mmus_LamA | *Mus musculus* | P48678 |
| Mmus_LamB1 | *Mus musculus* | P14733 |
| Mmus_LamB2 | *Mus musculus* | CAA38032 |
| Pcau_Lam | *Priapulus caudatus* | CAB43347 |
| Spur_LamB | *Strongylocentrotus purpuratus* | AAB34118 |
| Tcas_Lam | *Tribolium castaneum* | XP_972795 |
| Xlae_LamA | *Xenopus laevis* | P11048 |
| Xlae_LamB1 | *Xenopus laevis* | AAC31543 |
| Xlae_LamB2 | *Xenopus laevis* | AAC31544 |
| Xlae_LamL3 | *Xenopus laevis* | P10999 |
