# Supplementary material for: Characterization of lamin Mutation Phenotypes in Drosophila and Comparison to Human Laminopathies
Source: PLoS One. 2007 Jun 13;2(6):e532. doi: 10.1371/journal.pone.0000532 (PMC1885830; doi:10.1371/journal.pone.0000532)
Supplement: Figure S1 — Lamin phylogenetic comparisons. The three cladograms described in the text and presented in a condensed format in Fig. 5B are presented here in full. (0.17 MB PDF) [file pone.0000532.s002.pdf]

**Figure S1**

Lamin phylogenetic comparisons

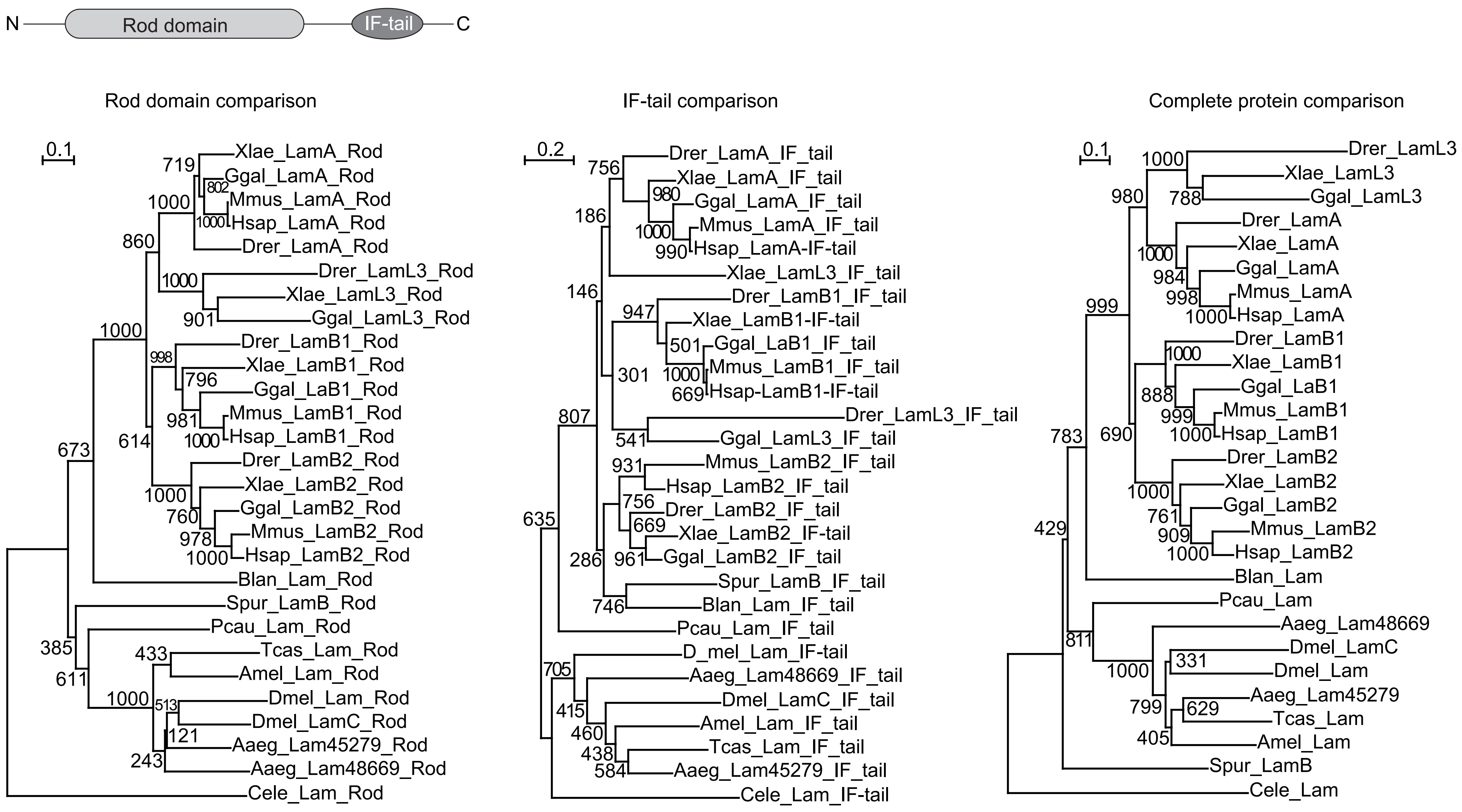


Bootstrap values are based on 1000 trials in all comparisons
